# Disseminated Cryptococcal Infection in Treatment-Naive Patients With Chronic Lymphocytic Leukemia (CLL)

**DOI:** 10.7759/cureus.59022

**Published:** 2024-04-25

**Authors:** Kakha Gujabidze, Subaha Akram Hussain, Stephanie Balint, Basel Saadeh, Sumeyra Yucelen, Bethel Shiferaw

**Affiliations:** 1 Internal Medicine, Saint Mary's Hospital, Waterbury, USA; 2 School of Medicine, Quinnipiac University, Hamden, USA; 3 Internal Medicine (Infectious Disease), Saint Mary's Hospital, Waterbury, USA

**Keywords:** cryptococcal neoformans, cll treatment, pulmonary cryptococcal infection, disseminated cryptococcus, chronic lymphocytic leukemia (cll)

## Abstract

This case report highlights a rare occurrence of disseminated cryptococcal infection in an elderly male with untreated chronic lymphocytic leukemia (CLL). Despite lacking prior treatment for CLL, the patient presented with respiratory symptoms and was found to have *Cryptococcus neoformans* in blood cultures. Prompt initiation of antifungal therapy was crucial, although central nervous system involvement was absent. The case underscores the importance of considering fungal infections in CLL patients, emphasizing the immunological vulnerabilities associated with the disease. Heightened awareness and early management are essential in addressing such opportunistic infections in this patient population.

## Introduction

Chronic lymphocytic leukemia (CLL) is the most common type of leukemia in adults in the United States [1]. It is thought of as a disease resulting from the accumulation of cells due to defective apoptosis. Overexpression of the antiapoptotic proteins B-cell lymphoma 2 (BCL2) and myeloid cell leukemia 1 (MCL1) was demonstrated in early investigations, along with the lack of proapoptotic proteins in CLL [2]. With unregulated proliferation comes consequences, such as immune dysfunction leading to increased susceptibility to infection.

Cryptococcosis is an infection caused by an encapsulated yeast - most commonly *Cryptococcus neoformans*. Disseminated infection is mostly seen in patients with HIV, reaching approximately 6% in patients with CD4 cell counts <100 cells [3]. Non-HIV-associated conditions include immunosuppression caused by corticosteroid therapy, chemotherapy or immunotherapy [4], and organ transplantation.

## Case presentation

Case history

**Figure 1:**
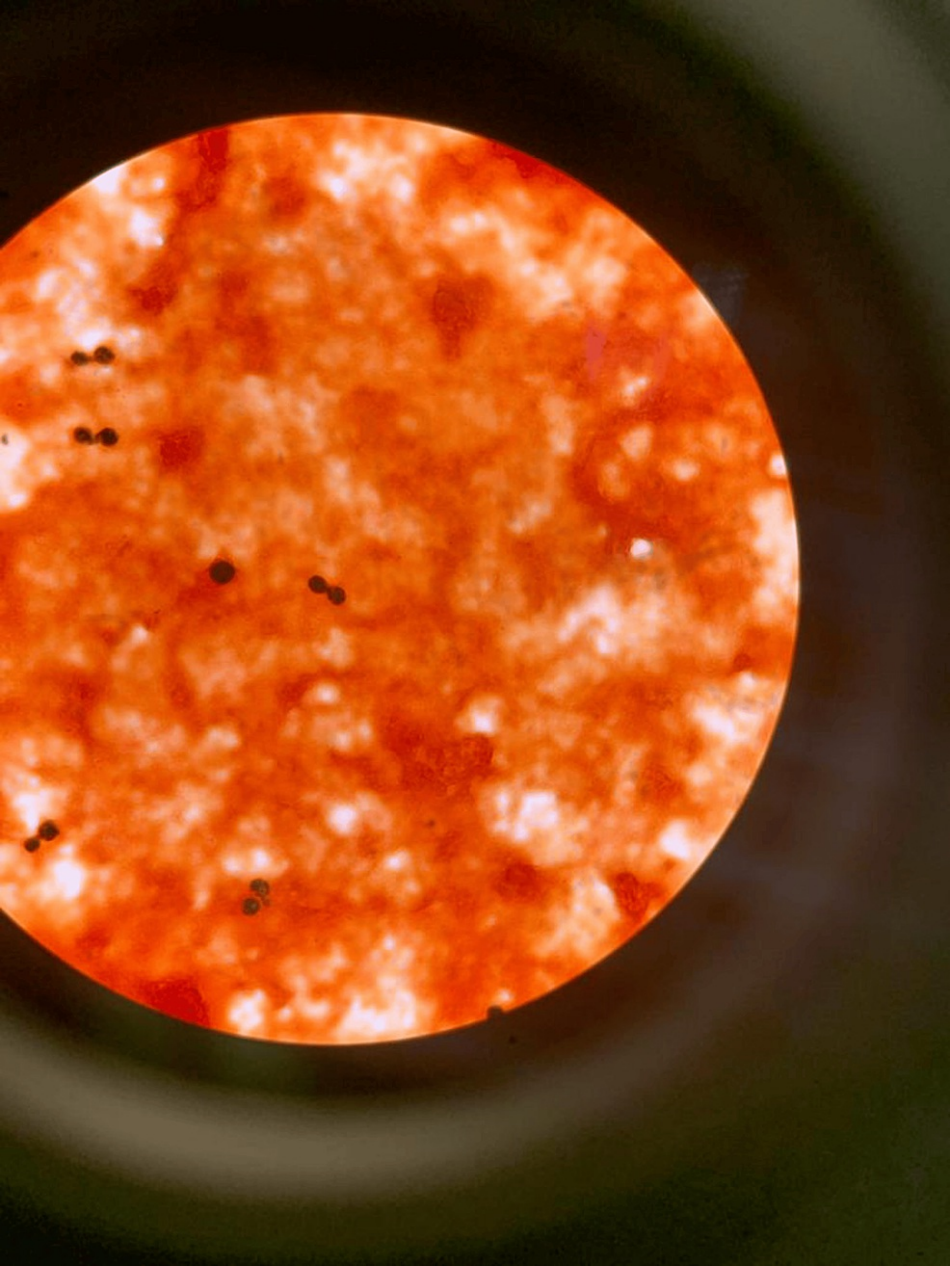
Cryptococcus neoformans from blood culture.

We present a case of a 65-year-old male with a past medical history of CLL, type 2 diabetes, and pulmonary embolism anticoagulated on apixaban who presented to the emergency department (ED) at the request of his primary care provider related to positive blood culture. *Cryptococcus neoformans* had grown in one out of two sets of blood cultures (Figure 1). The patient had been diagnosed with CLL in 2017, with 93% of cells showing Trisomy 12 and immunoglobulin heavy chain variable region (IGVH) being non-mutated. The patient had not received treatment for his CLL at the time of this case. 

The patient had originally presented to the hospital 1.5 months before the positive blood culture results. At that time, he reported shortness of breath for one month, with an increasing cough. Computed tomography angiography (CTA) showed a 1.3 cm nodular density in the right upper lobe. The plan was an admission, as the patient required five liters of oxygen to maintain oxygen saturation above 92%. However, the patient opted to leave against medical advice and was discharged on levofloxacin and a steroid taper. 

Two weeks later, the patient’s cough worsened, and he returned to the ED. CTA showed questionable right lower lobe pulmonary embolism, nonspecific diffuse ground-glass densities, and stable moderate mediastinal and right hilar lymphadenopathy. The patient was admitted for acute hypoxic respiratory failure and was started on ceftriaxone and azithromycin. On hospital day 2, the patient removed his intravenous access and requested to leave against medical advice. 

The following day, the patient returned to the ED hypoxic upon arrival. Subsequently, he was admitted for worsening shortness of breath. He was started on ceftriaxone, azithromycin, and solumedrol 40 mg intravenously (IV) every eight hours. He was transitioned to ceftazidime and vancomycin due to a history of levofloxacin failure as an outpatient. He was discharged on trimethoprim-sulfamethoxazole oral antibiotic and followed up with primary care and pulmonology as an outpatient. 

Three days later, the patient presented to ED with shortness of breath and was admitted for hypoxic respiratory failure. Pulmonology was consulted, and they interpreted the CT scan in the context of the patient's symptoms as indicating a pulmonary embolism in the right lower lung. As a result, he was started on apixaban 5 mg twice daily. Blood cultures were obtained during the hospitalization, and the patient was discharged on oral antibiotics as his condition improved. 

Ten days later, the hospital laboratory reported that *C. neoformans* was growing in one of two sets of the patient’s blood culture. The patient’s primary care doctor was notified, who, in turn, involved the pulmonologist and the infectious disease doctor. Cryptococcal antigen titer testing was performed, showing a serum level of 1:32. Imaging of the chest was ordered. As CT of the chest revealed worsening infiltrates, the patient was advised to return to the hospital for admission. 

Physical examination

Upon admission, his blood pressure was 123/73 mmHg, pulse was 104 beats per minute (bpm), respirations were 18 breaths per minute, temperature was 98.4 °F, and he was saturating 94% O_2_ on room air. He did not appear to be in distress, mucous membranes were moist, no murmurs were appreciated on auscultation, and his lungs revealed bilateral bibasilar crackles on examination. His skin was warm and dry with a brisk capillary refill, and he was alert and oriented to person, place, and time. 

His medications consisted of acetaminophen, albuterol, allopurinol, apixaban, aspirin, benzonatate, duloxetine, esomeprazole, finasteride, folic acid, gabapentin, fluticasone nasal spray, nystatin topical, triamcinolone topical, and a multivitamin. He lived at home with his wife, did not consume alcohol, and had quit smoking 40 years ago. Upon review of the systems, he complained only of fatigue. He had a past medical history of benign prostatic hyperplasia, type II diabetes with polyneuropathy, hypertension, hyperlipidemia, migraine headaches, and essential tremor. His surgical history included a cervical fusion, shoulder surgery, hand surgery, colonoscopies, and endoscopy. A chest X-ray was obtained on hospital day 1, revealing increased patchy densities in the bilateral lung bases, with increased nodularity in the left lung base. The findings were suspicious of pneumonia. 

**Figure 2:**
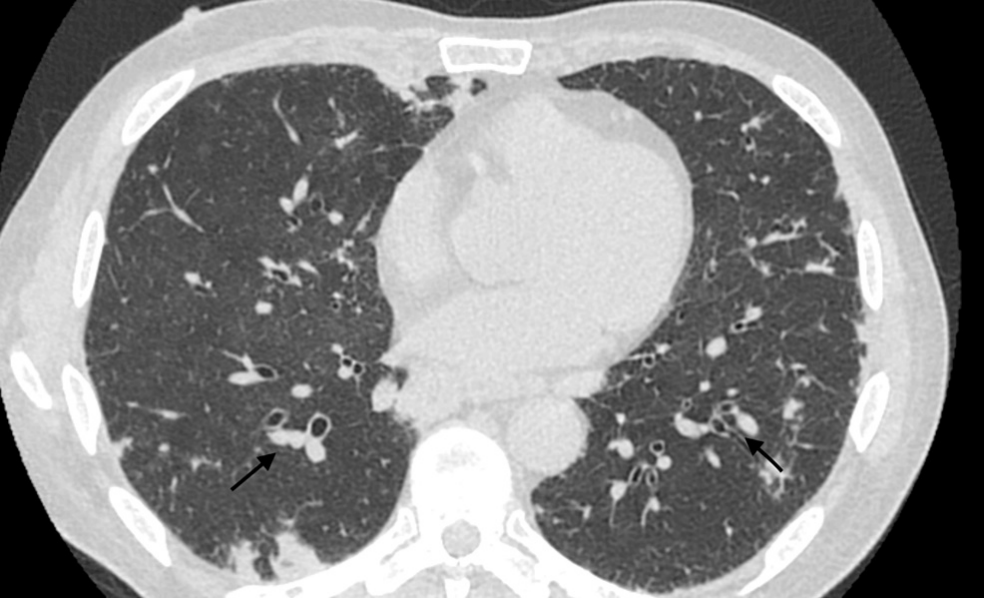
Initial computed tomography without contrast demonstrating bilateral nodular densities (black arrows), as well as mediastinal and hilar lymphadenopathy.

The subsequent CTA of the chest demonstrated bilateral consolidation and hilar lymphadenopathy, all of which had increased compared to the prior CTA and were concerning for pulmonary cryptococcosis (Figure 2).

Results of pathological examination

The patient underwent a lumbar puncture during the hospital stay to evaluate for central nervous system (CNS) involvement. Findings did not reveal any involvement - showing negative cryptococcal antigen, normal protein levels, and low WBC levels (2). The patient also underwent bronchoscopy, revealing erythematous airways in the right lung with scant purulent secretions. Bronchoalveolar lavage (BAL) revealed lymphocytic predominance, although no organism was identified on BAL samples.

Treatment plan

After admission, the patient was started on a combination of two antifungals due to disseminated cryptococcal infection - liposomal amphotericin 3 mg/kg daily as well as flucytosine 25 mg/kg oral every six hours for two weeks. The induction therapy was followed by consolidation therapy consisting of fluconazole 800 mg oral for eight weeks, with a plan of maintenance therapy of fluconazole 400 mg orally daily. The patient was discharged after two days of hospitalization with a plan to continue antifungal therapy via the PICC line and follow up with an infectious disease doctor, pulmonologist, and primary care physician.

## Discussion

We searched for articles using open-source resources (PubMed and Google Scholar) to find cases reporting disseminated cryptococcal infection in treatment-naive patients with CLL. Three other cases of disseminated cryptococcal infection were found [5-7]. In all cases, patients were male, with ages ranging from 60 to 84 years. Two of them had CNS involvement, and in one case, a lumbar puncture (LP) was not performed. All the patients had a subacute presentation, which is unlike the presentation commonly seen in HIV patients. Regarding morbidity, over one-half of the patients with CLL succumb to secondary infection [8]. Most commonly found infections are bacterial in origin and encompass *Streptococcus pneumoniae, Streptococcus aureus*, and *Haemophilus influenzae*. Viral reactivations are common and have been implicated in the progression of CLL itself as well. Fungal infections, especially disseminated ones are rare in untreated patients.

*C. neoformans* is a fungus pervasive in the environment but typically associated with bird feces and soil. The fungal spores are typically inhaled at which point they come into contact with innate immune cells. Macrovesicles play a role in transporting substances such as capsule precursors, enzymes, and melanin, from intracellular sites to the cell surface. The macrovesicles may play a role in the facilitation of *C. neoformans* penetrating the central nervous system [9]. In addition, it has been found in macrophages in vivo as a facultative intracellular organism. This ability has been called a *trojan horse mechanism*, allowing *C. neoformans* to exploit transendothelial pores to the blood-brain barrier leading to CNS infection [10].

Strains of *C. neoformans* that cause disease are known to inhibit the production of cytokines that would have activated macrophages (tumor necrosis factor [TNF] and interleukin-12 [IL-12]). By stimulating the production of IL-10, *C. neoformans* inhibits macrophage activation. Th17 cells are known to play a significant role in the adaptive immune response to extracellular fungi, while Th1-mediated immunity is associated with defense against intracellular fungi. In patients with CLL, both qualitative and quantitative immune defects are observed. Regarding T-cell function, it has been suggested that a decreasing number of CD4 cells as well as impairment of their function is seen in CLL and contributes to increased susceptibility to infection [11,12].

The critical role of CD8 cells in clearing cryptococcal infection has been demonstrated in the past as CD8 depletion by monoclonal antibodies resulted in decreased clearance of pulmonary cryptococcal infection [13]. Inverted CD4/CD8 ratios have also been seen in patients with CLL and have been associated with an increased risk of infections [14]. Hypogammaglobulinemia is present in one-quarter of newly diagnosed patients with CLL. Approximately one-quarter of patients with CLL with normal immunoglobulin G (IgG) at diagnosis will subsequently develop hypogammaglobulinemia on long-term follow-up [15]. The antibodies that B-cells produce in CLL have been described as target-restricted and were found to express a lower number of surface Igs than their normal counterparts, also leading to increased susceptibility to infection [16].

## Conclusions

In conclusion, this case study underscores the significance of recognizing and managing opportunistic infections, such as disseminated cryptococcosis, in patients with untreated CLL. Despite the rarity of fungal infections in this population, clinicians must remain vigilant for such complications to prevent morbidity and mortality. The absence of CNS involvement in this case is fortunate, but ongoing monitoring is essential due to potential complications. Understanding the immunological vulnerabilities associated with CLL is critical for early intervention and tailored management strategies. Ultimately, this report underscores the need for comprehensive care and heightened awareness of infectious complications in patients with CLL to optimize patient outcomes.
